# Sensitive Detection of *Staphylococcus aureus* by a Colorimetric Biosensor Based on Magnetic Separation and Rolling Circle Amplification

**DOI:** 10.3390/foods11131852

**Published:** 2022-06-23

**Authors:** Yutong Wang, Zhengzheng Wang, Zhongxu Zhan, Leina Yan, Lijun Wang, Hengyi Xu

**Affiliations:** 1 State Key Laboratory of Food Science and Technology, Nanchang University, Nanchang 330047, China; 402337519014@email.ncu.edu.cn (Y.W.); 412314919054@email.ncu.edu.cn (Z.W.); 2 Jiangxi Institute for Food Control, Nanchang 330047, China; 412314919024@email.ncu.edu.cn; 3 Jiangxi Institute for Drug Control, NMPA Key Laboratory of Quality Evaluation of Traditional Chinese Patent Medicine, Jiangxi Province Engineering Research Center of Drug and Medical Device Quality, Nanchang 330047, China; jxsyjs_ywk@126.com; 4 School of Food and Bioengineering, Xihua University, Chengdu 610039, China

**Keywords:** *Staphylococcus aureus*, DNA nanoflowers, vancomycin, magnetic separation, juice

## Abstract

*Staphylococcus aureus* (*S. aureus*) is a common foodborne pathogen that causes fever, vomiting, and other intestinal symptoms, and seriously affects human health and social safety. As a result, a reliable and sensitive detection technique for *S. aureus* must be developed. In this work, we proposed a sandwich assay on vancomycin functionalized magnetic beads (Van-MNPs) for *S. aureus* detection based on the specific binding between IgG and targets. The Van-MNPs were used as a tool for the separation of target bacteria. The biotin-modified IgG mediates binding between DNA nanoflowers (DNFs) and the target bacteria via interacting with streptavidin. The DNFs prepared by rolling circle amplification (RCA) were employed as a nano-container to enhance the capacity of biotins, and the streptavidin-horseradish peroxidase (SA-HRP) was loaded onto DNFs to catalyze the color change of TMB. Therefore, a colorimetric biosensor based on magnetic separation and rolling circle amplification was developed. The proposed methods for *S. aureus* detection showed a limit of detection (LOD) of 3.3 × 10^3^ CFU/mL and excellent specificity. The biosensor has a certain reference value for the detection of *S. aureus* in juice.

## 1. Introduction

Foodborne pathogens are one of the major causes of sickness and even death for humans [1]. *Staphylococcus aureus* (*S. aureus*), Vibrio parahaemolyticus, Escherichia coli, Pseudomonas aeruginosa, Listeria monocytogenes, Shigella, Salmonella, and other common foodborne pathogens could cause serious adverse effects to people’s health. *S. aureus* is one of the most frequent foodborne pathogens, capable of surviving in air, water, dust, and human and animal feces [2]. It can rapidly proliferate in a short period of time and produce enterotoxins. Many countries have strict detection limits on the number of *S. aureus* in foods [3]. Therefore, it is essential to establish a rapid, sensitive, and accurate assay for *S. aureus* detection.

At present, there are many strategies to detect *S. aureus*. The most common detection methods are based on culture [2] and molecular biological assays (such as PCR) [4]. The biosensor [5], surface plasmon resonance (SPR) [6], and quartz crystal microbalance [7] were gradually developed for the detection of *S. aureus*. However, these methods are susceptible to the matrixes, thus failing to achieve good performance [8]. Therefore, the samples are usually pretreated before the detection to reduce the interference of the matrixes.

Magnetic separation (MS) technology is a widely used sample pretreatment method for complex matrix processing; it can enrich and separate target or non-targeted pathogenic bacteria in complex matrix in a short time. Compared with traditional bacterial culture techniques, the magnetic separation technology takes less time and is easier to operate [9]. Vancomycin (Van) is a cheap and stable glycopeptide antibiotic, and could establish a five-point hydrogen bond with Gram-positive bacteria via D-alanyl-D-alanine. As a result, the vancomycin-modified magnetic nanoparticles (Van-MNPs) were used to trap Gram-positive bacteria and remove them from complicated food matrices. 

Researchers have discovered that DNA can be used as an excellent nano-scale material by forming secondary or tertiary nanostructures over the last few decades [10]. Researchers can use functional DNA nanomaterials to develop biosensors and medication delivery devices, for example [11,12]. To obtain the DNA nano-container, rolling circle amplification (RCA) has been applied in these studies [13]. The principle of RCA is that, under the function of the polymerase, the primer can bind with the circular template to generate a long repeat single-strand DNA (ssDNA) [14,15]. As the reaction time increases, the length of the ssDNA can also be expanded, and because of the magnesium ions in the buffer, the chains can be folded to form densely arranged DNA nanoflowers (DNFs) [16]. The obtained DNFs have a unique porous structure and are rich in Mg^2+^ [17,18]. They have high loading efficiency and stability, providing a range of interaction sites for physiologically active compounds such as enzymes, drugs, quantum dots, and so on [19,20]. Therefore, DNFs are often used as a nano-container for the transportation and release of biologically active substances [21].

We developed antibiotic-modified magnetic nanoparticles paired with biotin-loaded DNFs (AMS-DNFs) for the detection of *S. aureus* based on the findings of previous investigations. The Van-MNPs were used to capture Gram-positive bacteria from the food matrix. The system was then loaded with a large number of biotin-labeled DNFs, and the streptavidin modified horseradish peroxidase (SA-HRP) was introduced via the biotin-avidin system (BAS) [22,23]. Finally, after the enzyme substrate was added, HRP catalyzed the enzyme substrate, and a color change occurred [24]. The nanomaterial utilized in this work was simple, and the detection result can be observed by the naked eye without expensive and cumbersome equipment. The proposed AMS-DNFs method had a good specificity without interference from the complex food matrices, which showed a favorable application foreground of nanomaterials for detecting pathogenetic bacteria.

## 2. Materials and Methods

### 2.1. Material and Chemicals

Aladdin Industrial Corporation (Shanghai, China) provided N-Hydroxysuccinimide sodium salt (NHSS) and Vancomycin hydrochloride,1-(3-Dimethylaminopropyl)-3-ethylcarbodiimide hydrochloride (EDC). Carboxylated MBs (180 nm, 1 mg/mL) were provided by Allrun Nano Science and Technology Co. Ltd. (Shanghai, China). Sigma-Aldrich Chemical Co. Ltd. (Shanghai, China) provided bovine serum albumin (BSA). The agarose was obtained from Solarbio Technology Co., Ltd. (Beijing, China). The juice was obtained from the local supermarket. The DNA oligonucleotides (Table 1) prepared by TsingKe Biotech. Co. Ltd. (Beijing, China) were used. The dNTPs, phi29 DNA polymerase, and T4 DNA ligase were from SangonBiotech (Shanghai, China).

### 2.2. Apparatus

The nanomaterials were characterized to distinguish morphological structures by scanning electronic microscopy (SEM) (JSM-6701F, JEOL Ltd., Tokyo, Japan). The RCA products were characterized using a biomolecular imager (Imagequant LAS 500) and circular dichroism (MOS-450/AF Circular Dichroism Spectrometer). The colorimetric detection experiment was carried out using a microplate reader for data reading (Varioskan LUX).

### 2.3. Bacterial Culture and Counting

*Escherichia coli* O157:H7 ATCC43888, *Staphyloccocus aureus* CMCC26001, *Pseudomonas aeruginosa* CMCC10104, *Cronobacter* spp. CMCC45401, *Salmonella* Enteritidis ATCC13076, and *Listeria monocytogenes* ATCC13932 were used in this study and cultured with shaking in Luria-Bertani (LB) medium (37 °C, 180 rpm, 12 h). Then, 10-fold serial dilutions of bacteria were prepared in sterilized 0.01 M phosphate-buffered saline (PBS, pH = 7.4). After incubation at 37 °C for 12~18 h to enumerate, the number of viable bacteria was counted with agar plate count.

### 2.4. The Procedure for Synthesizing Van-MNPs

Van-MNPs were effectively produced in prior studies [10,26]. Firstly, 600 mg of carboxy groups-coated magnetic beads (MBs) were cleaned with 0.01 M of PBS (pH = 7) three times, and then activated by EDC (1.74 mg) and NHSS (1.95 mg) at 25 °C for 60 min. The MBs were cleaned by PBS with three times. Meanwhile, they were incubated for 120 min in PBS containing 7.2 mg of BSA. After eliminating the unreacted BSA with a magnetic field, BSA-MBs were obtained. Then, 45 mg of EDC and 15 mg of NHSS were combined with 72 mg of vancomycin and treated under dark conditions for 10 min to activate the carboxyl groups on the surface of vancomycin. Activated vancomycin was then added into the BSA-MBs solution and incubated for 120 min. Van-MNPs were finally collected using a magnet and stored at 4 °C for later use.

### 2.5. Preparation of DNFs

The DNFs used in this study were obtained by the RCA reaction. The DNA sequences used in this study are shown in Table 1. Firstly, 5 μL of primer and 6 μL of padlock were mixed and heated at 95 °C for 10 min, then the temperature was decreased to room temperature, allowing the formation of the round probe. The RCA template was prepared as follows: 11 μL circular probe solution, 10 × T4 DNA ligase reaction buffer (2 μL, 5 U/μL), ultrapure water, and the total reaction system was 40 μL. The ligation reaction was conducted (22 °C, 6 h), and then T4 DNA ligase was inactivated at 65 °C for 15 min. Next, the RCA reaction contained 2 μL phi29 DNA polymerase (10 U/μL), 20 μL phi29 DNA polymerase reaction buffer (10×), 28 μL dNTP (10 mM), 40 μL, RCA template, and 110 μL ultrapure water, and the total reaction volume was 200 μL. The mixture was incubated at 37 °C for 6~8 h. Then, RCA products had multiple copies of DNA that could bind with probe-biotin (100 μL, 100 nM) to form double-stranded DNFs [26].

### 2.6. The Principle of the AMS-DNFs Strategy

**Scheme 1:**
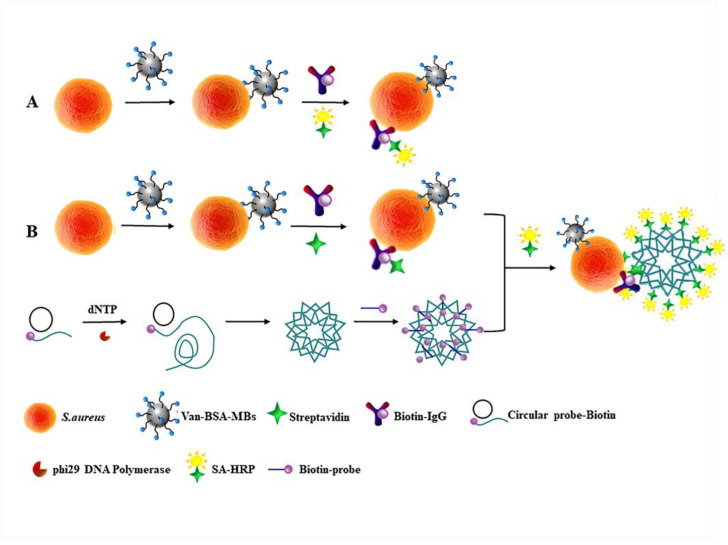
The scheme of the AMS-DNFs detection method. (**A**) The antibiotic magnetic separation AMS-DNFs amplification colorimetric detection method and (**B**) common colorimetric detection method for *S. aureus*.

**Figure 1:**
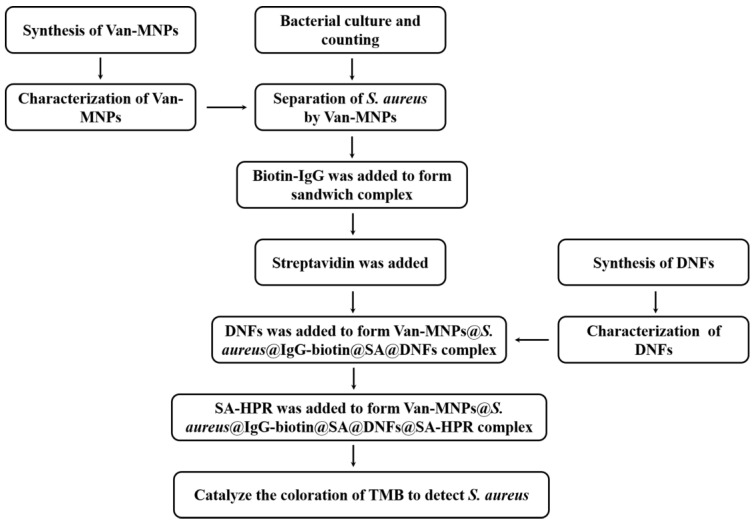
Flow chart of experimental steps.

The AMS-DNFs approach for identifying *S. aureus* was established in this work. Scheme 1 and Figure 1 depict the measurement principle. Firstly, 100 μL of Van-MNPs (1 mg/mL) and 100 μL of various concentrations of *S. aureus* were incubated at 37 °C for 50 min. Next, the mixtures were washed thrice with PBS under an external magnetic field. The IgG-biotin (5 nM) was added into the complex of MBs–bacteria and incubated (37 °C, 1 h). After removing excess IgG-biotin by washing three times with PBS, the experiment was divided into two groups—one was the common colorimetry assay (without DNFs) to detect *S. aureus* and the other was the DNFs signal amplification colorimetry to detect *S. aureus*. 

Without DNFs, the experiment was conducted as follows: 50 μL of SA-HRP was added in complex, and linked with MNPs@*S. aureus*@IgG-biotin through the interaction of biotin and streptavidin at 37 °C for 30 min. After washing thrice with PBS to remove excess SA-HRP, 50 μL of enzyme substrates were added and incubated (10 min, 37 °C). The reaction was stopped by adding H_2_SO_4_ (50 μL, 10% (*v*/*v*)) and the absorbance was measured at 450 nm using a multimode microplate reader.

The experiment with DNFs was treated as follows (Figure 1): 1.5 μg of SA was added and linked with the complex at 37 °C for 30 min through the interaction of biotin and SA to form Van-MNPs@*S. aureus*@IgG-biotin@SA. After washing thrice with PBS to remove excess streptavidin, 100 μL of DNFs were added in complex and incubated with Van-MNPs@*S. aureus*@IgG-biotin@SA at 37 °C for 30 min. After washing thrice with PBS to eliminate extra DNFs under an external magnetic field, SA-HRP was added to the system at 37 °C for 30 min. Finally, the Van-MNPs@*S. aureus*@IgG-biotin@SA-HRP@DNFs were washed thrice with PBS, and then 50 μL of enzyme substrate was added and incubated at 37 °C for 10 min. After the reaction was completed, the absorbance of the solution was measured according to the above method.

### 2.7. Specificity Analysis

Four Gram-negative bacteria and one Gram-positive bacterium, as well as Mix1 (five non-objected bacteria) and Mix2 (the five non-objected bacteria+ target bacteria), were chosen to test the specificity of the DNFs assay. The experiment was verified using DNFs and standard colorimetry.

### 2.8. Detection of S. aureus in Juice Sample 

*S. aureus* were mixed with the juice samples (100 μL of bacteria mixed with 900 μL of juice) and the final concentration of *S. aureus* was 10^2^ to 10^8^ CFU/mL. The juice samples (1 mL) were then mixed with 100 L Van-MNPs and reacted at 37 °C for 50 min with shaking. The samples were stood in external magnetic field for 3 min and washed with PBS three times, the Van-MNPs@*S. aureus* were resuspended with PBS (500 μL). IgG-biotin (10 μL) was added and reacted with shaking (37 °C, 1 h). After washing thrice, the Van-MNPs@*S. aureus*@IgG-biotin were resuspended in PBS (300 μL). Then, 1.5 μg of SA was added and linked with the complex at 37 °C for 30 min through the interaction of biotin and SA to form Van-MNPs@*S. aureus*@IgG-biotin@SA. Next, 100 μL of DNFs were added in complex and incubated with Van-MNPs@*S. aureus*@IgG-biotin@SA-HRP@DNFs for 30 min at 37 °C. SA-HRP was added to the systems at 37 °C for 30 min. Finally, the Van-MNPs@*S. aureus*@IgG-biotin@SA-HRP@DNFs system was washed thrice with PBS, after which 50 μL of TMB color development solution was added and it was reacted at 37 °C for 10 min. After the reaction was completed, the absorbance of the solution was measured according to the above method.

### 2.9. Statistical Analysis

The experimental data of this study were displayed as the mean ± SD, and SPSS (Version 22) software was used for the statistical analysis of the data. The analysis of the difference between groups was carried out using one-way ANOVA. Specially, at least three independent parallel experiments were conducted in each group to ensure the authenticity of the reported data.

## 3. Results and Discussion

### 3.1. Strategy of AMS-DNFs for the Detection of S. aureus 

The AMS-DNFs assay was proposed and applied in the detection of *S. aureus* (showed in Scheme 1). Firstly, Van-MNPs were prepared by following previous strategies [10]. Van-MNPs were used to identify and enrich *S. aureus* from the juice sample. To achieve the specific detection of *S. aureus*, biotin-IgG and SA were added because the Fc of IgG may bind selectively to *S. aureus* protein A. Meanwhile, DNFs loaded with biotin were prepared by RCA reaction. The MNPs-Van@*S. aureus*@IgG-biotin@SA@DNFs complex was formed under the action of the SA-biotin system. After adding SA-HRP, the MNPs-Van@*S. aureus*@IgG-biotin@SA@DNFs@SA-HRP complex was formed. Finally, SA-HRP was added to catalyze the coloration of TMB to detect *S. aureus* with DNFs signal amplification or common colorimetry.

### 3.2. Characterization of Antibiotic-MBs and DNFs

**Figure 2:**
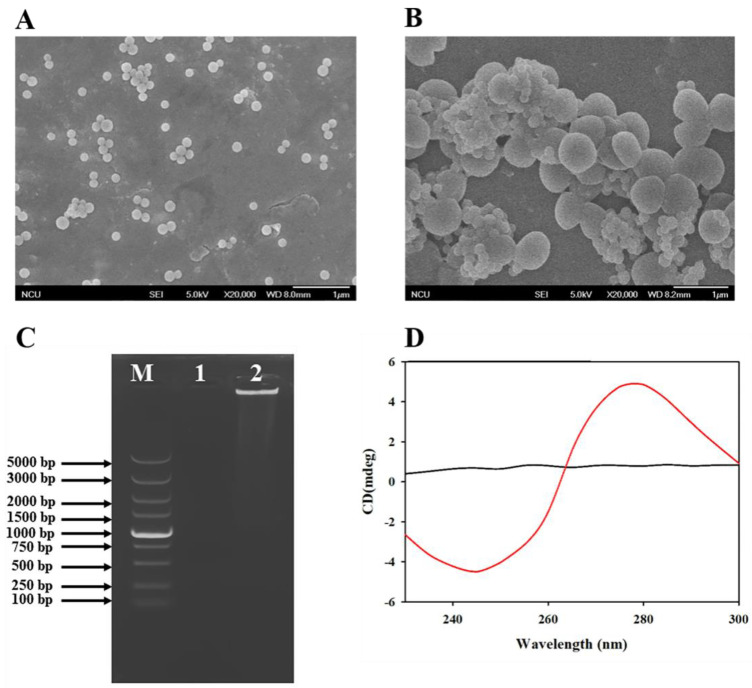
The SEM images of (**A**) Van-MNPs, (**B**) *S. aureus* captured by Van-MNPs, and (**C**) 1.5% agarose gel electrophoresis images of the RCA products. Lane M: DNA marker, Lane 1: the padlock without primer, Lane 2: padlock and primer. (**D**) Circular dichroism spectroscopy for the assay.

The images of Van-MNPs and the capture of *S. aureus* by Van-MNPs were validated by SEM. The image of Van-MNPs is presented in Figure 2A. The image shows that the shape of Van-MNPs was round and uniform, and Figure 2B reveals that the antibiotic magnetic material could link with the cell wall of *S. aureus*. Each bacterial surface had multiple magnetic materials, indicating that the Van-MNPs could capture *S. aureus*. 

Here, 1.5% agarose gel electrophoresis was used to prove the success of the RCA reaction. When primer and padlock were present in Figure 2C, a new brilliant band appeared in the gel hole, indicating that primer could start the RCA reaction. Besides, to further verify the occurrence of RCA, this experiment carried out the characterization of circular dichroism in Figure 2D. The results confirmed that there was a clear peak at 280 nm. However, there was no peak at 280 nm when missing a particular reagent.

### 3.3. Optimization of Colorimetry Experimental Conditi

The experimental settings for both the ordinary colorimetric detection and the DNFs signal amplification colorimetric approach were also tuned to produce the optimum detection signal. 

**Figure 3:**
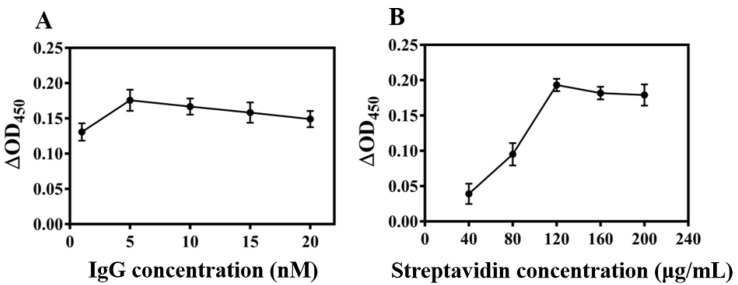
The common colorimetric detection method parameters’ optimization. (**A**) Optimization of IgG-biotin concentration. (**B**) Optimization of SA-HRP concentration. The concentration of *S. aureus* used was 3.3 × 10^7^ CFU/mL. The error bars represent the standard deviation of three measurements.

The amounts of IgG-biotin and SA-HRP were optimized without DNFs experiment. The optimal amount of IgG-biotin was selected using the difference between the positive value and the blank value. As illustrated in Figure 3A,B, the highest ∆ODs were obtained when the concentrations of IgG-biotin and SA-HRP were at 5 nM and 120 μg/mL, respectively. The concentration of bacteria was 10^7^ CFU/mL and the amount of Van-MNPs (1 mg/mL) was 100 μL. 

**Figure 4:**
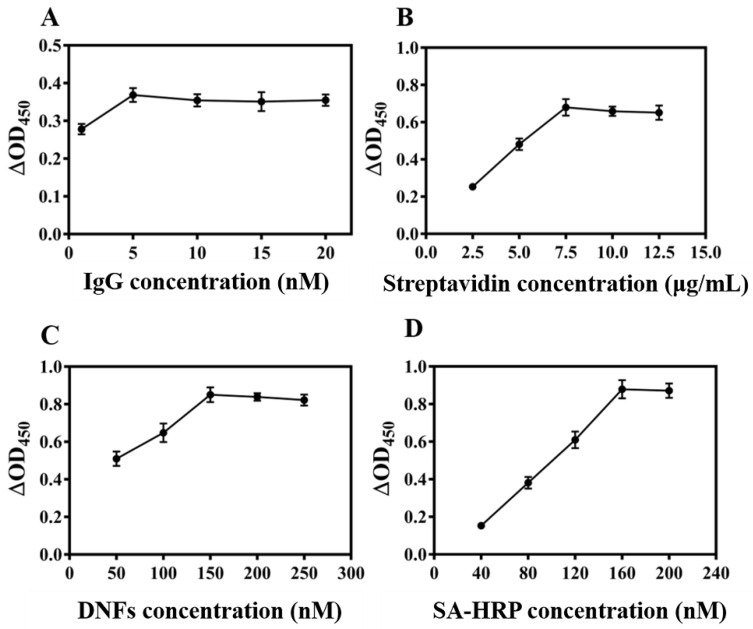
The AMS-DNFs amplification colorimetric method parameters’ optimization. (**A**) Optimization of biotin-IgG concentration. (**B**) Optimization of SA concentration. (**C**) Optimization of DNFs concentration. (**D**) Optimization of SA-HRP concentration. The concentration of *S. aureus* was 3.3 × 10^5^ CFU/mL. The error bars represent the standard deviation of three measurements.

The colorimetric detection method with DNFs signal amplification involved five parameters. As shown in Figure 4A, when the concentration of IgG-biotin was 5 nM, the ∆OD reached the maximum. The amount of SA also played a key role in this assay. According to the optimization experiment, when the streptavidin was 7.5 μg/mL in Figure 4B, the ∆OD was the highest. In addition, the amount of DNFs was also optimized. By considering economic factors and the strength of the detection signal, the optimal concentration of DNFs was 150 nM in Figure 4C. In addition, the concentration of SA-HRP has to be tuned to minimize excessive enzyme waste. As shown in Figure 4D, the optimal concentration of SA-HRP was 160 μg/mL. All of the optimizations of this experiment were performed at the concentration of bacteria of 10^5^ CFU/mL and volume of 100 μL Van-MNPs (1 mg/mL).

In conclusion, this study was carried out according to the following conditions: the concentrations of IgG-biotin and SA-HRP were at 5 nM and 120 μg/mL, respectively; the amount of Van-MNPs (1 mg/mL) was 100 μL; and the concentration of SA, DNFs, and SA-HRP was 7.5 μg/mL, 150 nM, and 160 μg/mL, respectively.

### 3.4. Specificity of Two Colorimetric Experiment 

**Figure 5:**
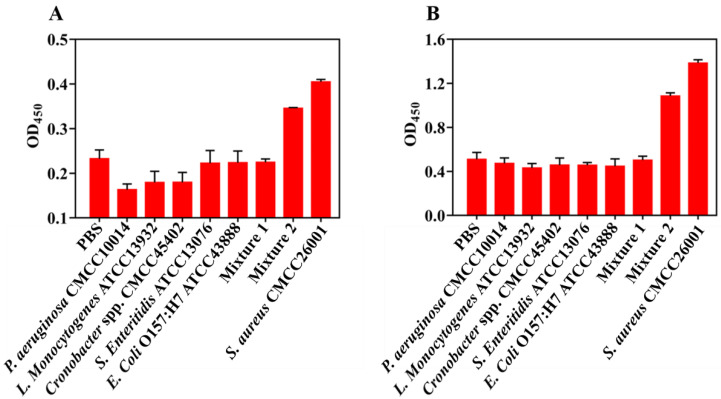
The specificity of common colorimetric and AMS-DNFs detection method. (**A**) The specificity of common colorimetric detection method for *S. aureus* and non-target bacteria. (**B**) The specificity of the AMS-DNFs amplification colormetric detection method for *S. aureus* and non-target bacteria. The concentration of non-target bacteria was 10^7^ CFU/mL.

The specificity of antibiotic magnetic separation combined with colorimetry and DNFs amplification colorimetry was proven to verify the viability of two colorimetry ex-periments. Van, being a broad-spectrum antibiotic, was able to recognize a variety of Gram-positive bacteria, while IgG served as a secondary recognition molecule, allowing *S. aureus* to be identified more precisely. Therefore, in this study, four Gram-negative bacteria and one Gram-positive bacteria were selected for specific experiments. The PBS was used as a negative control. As shown in Figure 5A,B, the absorbance responses from *C. sakazakii*, *S. Enteritidis*, *E. coli*, and *P. aeruginosa* and Mix1 were similar to the control group, but the absorbance of target bacteria and Mix2 was obviously higher than control group. The results showed that both the common colorimetric method and the DNFs signal amplification colorimetry had good specificity.

### 3.5. Evaluation of AMS-DNFs Assay in Juice Samples

**Figure 6:**
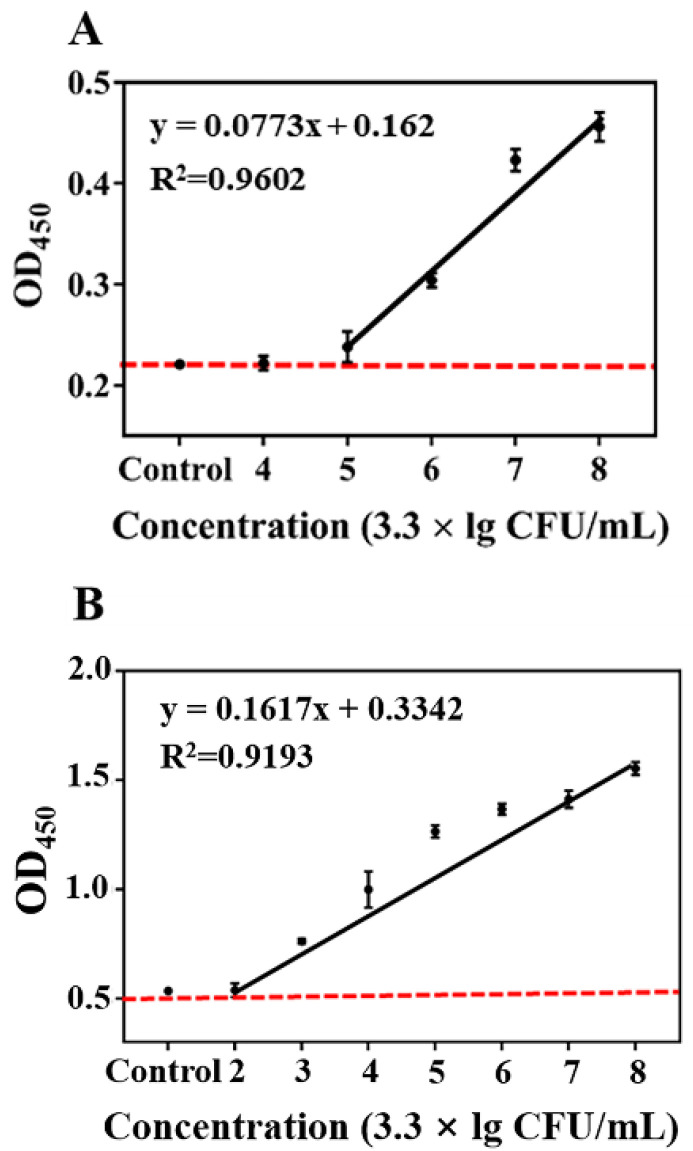
(**A**) The LOD and calibration curve of the common colorimetric detection method for *S. aureus* in pure culture. (**B**) The LOD and calibration curve of the AMS-DNFs amplification colormetric detection method for *S. aureus* in pure culture.

**Figure 7:**
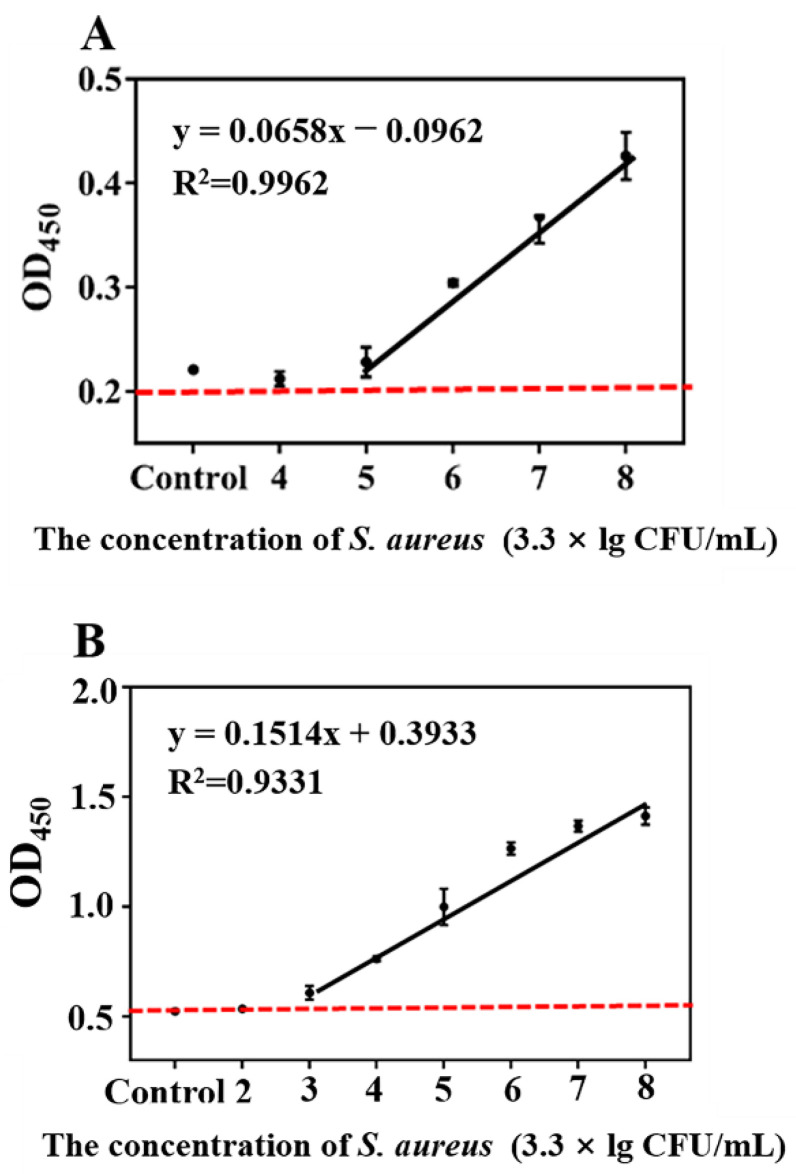
(**A**) The LOD and calibration curve of the common colorimetric detection method for *S. aureus* in juice samples. (**B**) The LOD and calibration curve of the AMS-DNFs amplification colorimetric detection method for *S. aureus* in juice samples.

To assess the effect of the common colorimetry and the AMS-DNFs assay, *S. aureus* were added to juice samples, as well as PBS, and they were tested under optimal conditions. As shown in Figure 6 and Figure 7, with the concentration of bacteria increasing, the absorbance of both the common colorimetry and AMS-DNFs signal amplification colormetry gradually increased in the PBS buffer and juice samples. Bacterial concentration and 450 nm absorbance have a positive connection, according to these data. A linear correlation of the common colorimetric and the concentration of *S. aureus* in Figure 6A was recorded at y = 0.0773x + 0.162 (R^2^ = 0.9602), where y is absorbance at 450 nm and x is the concentration of *S. aureus* (10^5^ CFU/mL to 10^8^ CFU/mL). Besides, in Figure 6B, a linear correlation of the AMS-DNFs assay between the concentration of *S. aureus* was recorded at y = 0.1617x + 0.3342 (R^2^ = 0.9193), where y is absorbance at 450 nm and x is the concentration of *S. aureus* (10^3^ CFU/mL to 10^8^ CFU/mL). The LOD in PBS of AMS-DNFs assay and common colorimetry was 3.3 × 10^2^ CFU/mL and 3.3 × 10^5^ CFU/mL based on NC + 3SD, respectively. In the juice sample, the common colorimetry and AMS-DNF also had a good linear relationship. In Figure 7A, in the concentration range of 3.3 × 10^5^ CFU/mL to 3.3 × 10^8^ CFU/mL, the linear correlation of common colorimetry was good, where y = 0.0658x − 0.0962 (R^2^ = 0.9962). In Figure 7B, the linear correlation of the AMS-DNFs assay was good in the concentration range from 3.3 × 10^3^ CFU/mL to 3.3 × 10^8^ CFU/mL, where y = 0.1514x + 0.3933 (R^2^ = 0.9331). The LODs in juice samples of the AMS-DNFs assay and common colorimetric were 3.3 × 10^3^ CFU/mL and 3.3 × 10^5^ CFU/mL, respectively.

## 4. Conclusions

We developed a method for the detection of *S. aureus* by Van-MNPs combined with DNFs signal amplification colorimetry. Van-MNPs were effectively synthesized and utilized to efficiently enrich *S. aureus* in a short amount of time. In comparison with traditional colorimetry, DNFs prepared by RCA could load a significant quantity of horseradish peroxidase to amplified detection signals. Common colorimetry detection had a linear correlation between 10^5^~10^8^ CFU/mL and the visual detection limit was 3.3 × 10^5^ CFU/mL. The AMS-DNFs signal amplification colorimetric method had a linear correlation between 10^3^~10^8^ CFU/mL and the LOD was 3.3 × 10^3^ CFU/mL. The AMS-DNFs signal amplification colorimetric method was feasible for the sensitive and visible detection of *S. aureus*. The reaction conditions were moderate; the cost was inexpensive; and it was not necessary to use special instruments, which was very convenient and had a certain practical application. This method had certain guiding significance for establishing AMS combined with DNFs nano-containers for the detection of pathogenic bacteria.

## Figures and Tables

**Table 1:** The sequences of primers and probes.

Name	Sequence (5′–3′)	Ref.
RCA primer	Biotin-TACCCGGTTGATAAAAAAAATTGGTTATGTGGCTGATGTTGC	[25]
Padlock DNA	PO_4_-CACATAACCAAAAACCCGAAGAGAAGGAAGAG AAAGCAACATCAGC	[25]
Biotin probe 3	Biotin-CCCGAAGAGAAGGAAGAGAAA	[25]

## Data Availability

The data presented in this study are available on request from the corresponding author.
